# RGFA: powerful and convenient handling of assembly graphs

**DOI:** 10.7717/peerj.2681

**Published:** 2016-11-08

**Authors:** Giorgio Gonnella, Stefan Kurtz

**Affiliations:** Zentrum für Bioinformatik, Universität Hamburg, Hamburg, Germany

**Keywords:** GFA format, Sequence assembling, Assembly graph, Software library, Graphical Fragment Assembly, Graph transformation

## Abstract

The “Graphical Fragment Assembly” (GFA) is an emerging format for the representation of sequence assembly graphs, which can be adopted by both de Bruijn graph- and string graph-based assemblers. Here we present RGFA, an implementation of the proposed GFA specification in Ruby. It allows the user to conveniently parse, edit and write GFA files. Complex operations such as the separation of the implicit instances of repeats and the merging of linear paths can be performed. A typical application of RGFA is the editing of a graph, to finish the assembly of a sequence, using information not available to the assembler. We illustrate a use case, in which the assembly of a repetitive metagenomic fosmid insert was completed using a script based on RGFA. Furthermore, we show how the API provided by RGFA can be employed to design complex graph editing algorithms. As an example, we developed a detection algorithm for CRISPRs in a de Bruijn graph. Finally, RGFA can be used for comparing assembly graphs, e.g., to document the changes in a graph after applying a GUI editor. A program, GFAdiff is provided, which compares the information in two graphs, and generate a report or a Ruby script documenting the transformation steps between the graphs.

## Introduction

The advent of the next generation sequencing technologies was accompanied by the development of analysis tools, able to cope with the large datasets and the peculiarities of different sequencing platforms. An important class of sequence analysis tools are sequence assemblers, which aim at assembling a set of sequencing reads into a complete sequence.

Most assembly programs are based on graph representations of common substrings of the sequencing reads. Some popular assembly programs, such as Velvet (Zerbino & Birney, 2008) and SPAdes (Bankevich et al., 2012) employ a *de Bruijn graph*. In this graph, each *k*-mer *w* in the sequence reads corresponds to an edge in the graph connecting those nodes, representing respectively, the two (*k* − 1)-mers of *w*. Other assemblers, such as SGA (Simpson & Durbin, 2012) and Readjoiner (Gonnella & Kurtz, 2012) employ a *string graph*. This is a representation of the set of strings compatible with the set of sequencing reads and their overlaps. It can be obtained from an *overlap graph*, in which each read is represented by a node, and each edge stands for a suffix-prefix match of a pair of reads.

The final output of an assembler program is, in most cases, a collection of contig sequences, obtained by traversing the assembly graph. However, the assembly graph itself offers more information than the set of contigs: it contains, in a compact form, all information about sequence similarities between the sequence reads. As a consequence, all possible solutions for assemblies involving sequence repeats can be derived from the assembly graph. Sequence polymorphisms, repeats and lack of coverage in the sequence data lead to characteristic signatures in the graph and a careful analysis of these signatures may provide an understanding of, e.g., why the assembler traversing the paths in the graph does not deliver a complete genome sequence.

Manual editing of the graph is often necessary to improve or finish the assembly. For example, certain paths may be selected based on additional information and decisions about the orientation of a given sequence may be made. Although assemblers often output the assembly graph to file, an obstacle to the development of tools for handling those graphs has been the lack of a common file format.

In 2014 the “Graphical Fragment Assembly” (Li, 2014) was introduced as a common format. It is compatible to both de Bruijn graphs and string graphs. Subsequently, a collaborative open project has developed the initial draft into a full specification proposal (GFA Format Specification Working Group, 2016). The proposed GFA specification has been adopted by different software packages; however, support for the manipulation of GFA graphs in scripting languages is currently very limited (see ‘Discussion’).

In this work, we present RGFA, an implementation of the GFA specification in the scripting language Ruby. It offers an API to parse, edit and write GFA files, complying with the proposed standard. The RGFA library is complemented by the RGFATools. These exploit the flexibility of the GFA format, by employing their own custom fields and own naming conventions convenient for more advanced graph manipulation algorithms.

The API provided by RGFA was designed to facilitate easy implementation of custom analysis pipelines. The readability of code based on RGFA well supports the documentation of ad-hoc developed editing steps applied to assembly graphs. Such a documentation is sometimes required to allow reproducibility of an assembly which has been manually finished. Another use case of RGFA is the rapid prototyping of new graph editing algorithms. We give examples of these scenarios by providing use cases of RGFA for assembling repetitive fosmid inserts and for detecting Clustered Regularly Interspaced Short Palindromic Repeats (CRISPRs) in de Bruijn graphs.

RGFA and RGFATools are available at http://github.com/ggonnella/rgfa.

## Methods

### RGFA and RGFATools

RGFA has been developed using Ruby, version 2.0 and follows the conventions and format specified by the packaging system Rubygem.

The RGFA package provides an implementation of the “Graphical Format Assembly” (GFA) format in Ruby. In particular, the proposed GFA specification (GFA Format Specification Working Group, 2016) (last commit on May 17. 2016) is fully supported. RGFA contains methods to construct a GFA graph, read a graph from file, validate and edit it and write it back to file. It also allows simple graph manipulation, limited to operations which do not make any assumption on the graph content and do not define any custom fields.

RGFATools is implemented as a separate part of the RGFA package, which can be optionally required if needed. It provides further methods and scripts to manipulate, simplify and compare graphs. In contrast to the main RGFA code, which is generic, some assumptions are made, e.g., requiring the presence of count information on segments. Furthermore, RGFATools also defines several optional custom fields required by its operations. This contrast is the main reason for splitting the software into two parts.

### GFA graphs

A GFA file specifies records representing the GFA graph. Sequences are represented by nodes, named *segments* (record type S). The segments are connected by different kinds of edges both representing suffix-prefix matches (SPMs) of pairs of segments; *containments* (record type C) represent SPMs in which one segment is completely contained in the other and *links* (record type L) represent SPMs for which this is not true. Furthermore, the GFA file can contain generic information in *header* records (type H) and paths in the graph can explicitly be represented by dedicated *path* records (type P).

The GFA graph was implemented in the RGFA class of RGFA. It is comprised of a collection of RGFA::Line objects, as described below. Segments and paths are stored in hash tables, which allow a simple and efficient lookup by record name. The graph traversal is made possible by references which connect links/containments and segments, in both directions.

The class RGFA provides a method info to obtain basic topology information, such as the number of connected components and of the number of dead-ends, as well as sequence statistics, such as the total, longest, shortest, average and N50 segment sequence lengths.

### The format of GFA lines

Each line in a GFA file specifies a record consisting of tab-separated fields. The first field is always required (empty lines can be ignored) and it consists of a single capital letter specifying the record type. Depending of the record type, further fields are required. Past the required fields, optional fields (also denoted tags) can follow.

The record type and the other required fields are defined by the column they occur in. Optional fields consist of field name, data type and value. The field name consists of two letters or one letter and one number: upper case letters are reserved for optional fields pre-defined in the GFA specification, while applications can define custom fields using lower case letters for their name. The available data types for optional fields are A (single characters), i (signed integers), f (floating points numbers), Z (strings), J (JSON data), H (byte arrays) and B (numeric arrays).

Lines of the GFA format are implemented in RGFA by the class RGFA::Line. For each record type, a subclass of RGFA::Line is defined. This provides a list of required fields and predefined optional fields, as well as methods relevant only to a specific record type. The data type for each field is validated using the regular expression provided in the GFA specification. For optional fields, the field name is also validated according to the above mentioned criteria. Methods for setting and getting field values and creating new optional fields are dynamically generated.

When accessed, the value of a field is converted from or into an appropriate Ruby class. This applies to required fields, whenever possible, and to optional fields of type i (Integer), f (Float), B (Array of Integer or Float) and J (Array or Hash).

The class GFA provides methods to add objects derived from header, segment, link, containment and path lines to a graph. Once a graph has been completely read or constructed, references to the segments in links, containments and paths can be validated. If a segment is deleted, the deletion is cascaded (by default) to all links, containments and paths referring to it. If a segment or path is renamed, all references to it are updated.

### Double strand sequences, segment orientation and segment ends

As the segments refer to DNA sequences, it always represents a sequence and its reverse complement at the same time. In all references to a segment in GFA (stored in the fields *from* and *to* of links and containments and in the field *segment names* of paths) the segment names are always accompanied by a flag specifying the orientation of the segment in the current context.

Some assembly graphs, such as the original string graph as defined by Myers (2005), explicitly handle the two ends of a sequence as related but separate entities. Thereby, the B and E ends of a sequence can be viewed as imaginary points located, respectively, before the beginning and after the end of the sequence in its forward orientation.

In the GFA specification, there is no explicit concept of segment ends. However, in order to traverse and simplify the graph, it is useful to explicitly consider the segment ends. For this reason, RGFA provides methods which allow the user to retrieve the links between two specific segment ends or all links of a given segment end.

A conversion from the internal links representation is obtained as follows. The links of the B-end of a segment are all links either *from* the segment in *reverse* orientation, or links *to* the segment in *forward* orientation. The links of the E-end of a segment are all links *from* the segment in *forward* orientation or *to* the segment in *reverse* orientation.

### Read count, coverage and copy number

The GFA specification defines optional fields for storing counts, namely the number of reads (RC), *k*-mers (KC) or fragments (FC), (all denoted by *c* in this context) supporting a particular segment. This information can either be provided directly by the assembler, or obtained by mapping the sequencing reads to the segment sequences.

RGFA provides a method for computing the coverage of a segment *s*. The average length of reads or fragments or the *k*-mer count, all denoted by ℓ in this context, can be provided to compute a more accurate coverage for short segments as follows: $\mathit{coverage}(c,s,\ell )= \frac{c}{{|}s{|}-\ell +1} $.

RGFATools provide a method which allows to compute, based on the coverage, an estimated copy number for each segment. The current implementation requires the user to provide a coverage value *scov* of single copy segments. A minimal coverage *mincov* can also be provided. By default *mincov* = 0.25⋅*scov*. Then for each segment *s* of coverage *cov*, the copy number *cn*(*s*, *cov*) is defined by

\begin{eqnarray*}cn(s,\mathit{cov})= \left\{ \begin{array}{@{}ll@{}} \displaystyle 0\hspace*{10.00002pt}&\displaystyle \text{if}~\mathit{cov}\lt \mathit{mincov}\\ \displaystyle 1\hspace*{10.00002pt}&\displaystyle \text{if}~\mathit{mincov}\leq \mathit{cov}\lt \mathit{scov}\cdot 1.5\\ \displaystyle \left\lfloor \frac{\mathit{cov}}{\mathit{scov}} +0.5 \right\rfloor \hspace*{10.00002pt}&\displaystyle \text{otherwise}. \end{array}. \right. \end{eqnarray*}

### Segment multiplication

RGFA provides a method to clone a given segment *s*. The method requires the user to specify a multiplication factor *m* ≥ 2. It replaces *s* by *m* segments (termed here *copies of *s**) identical to *s*, except for the count tag values, which are divided by *m*.

By default, the method duplicates all links of *s* in each of the copies. However, this is not always ideal. For example, consider the situation where an end of a segment *s*_1_ is connected to two segments *s*_2_ and *s*_3_, and the copy number of *s*_1_ is double that of *s*_2_ and *s*_3_. In this case, it is useful to distribute the links of *s*_1_ among the copies ${s}_{1}^{{^{\prime}}}$ and ${s}_{1}^{{^{\prime\prime}}}$ of *s*_1_, so that the link to *s*_2_ is assigned to ${s}_{1}^{{^{\prime}}}$ and the link to *s*_3_ is assigned to ${s}_{1}^{{^{\prime\prime}}}$. For this reason, RGFATools extends the multiplication operation, providing a links distribution feature, described in the next section.

Furthermore, RGFATools provides origin tracking for the multiplication operation, introducing a custom string field or (origin). If *s*.or is not yet set, then *s*.or is set to *s*.name, the name of *s*. The value of *s*.or is then copied to all clones.

### Distribution of links among the copies of a segment

Let *S* be the set of segments cloned from *s*, which replace *s* after multiplication. Let *L* be the set of links of one of the ends of *s*. RGFATools provides an optional extension of the multiplication operation, which can be applied if the segments in *S*, as well as all segments reachable from them have the copy number 1 after multiplication. In this case, RGFATools distributes *L* on *S* as follows.

Let *m* be the multiplication factor (number of copies of *s* after multiplication). Heuristic criteria are applied to select the end of *s* from which *L* is taken to be distributed on *S*. The criteria aim at eliminating as many links as possible, without loosing any information. The process of distributing *L* on *S* is then performed as follows. The segments *S* = {*S*_1_, *S*_2_, …, *S*_*m*_} are processed one after the other. Let *n* = |*L*| and *L* = {*L*_1_, *L*_2_, …, *L*_*n*_}. In the *i*-th iteration, links are assigned to *S*_*i*_ as follows. If *n* ≤ *m*, then *L*_*i*_ is assigned to *S*_*i*_. The last *m* − *n* elements of *S*, if any, will remain without a link.

If *n* > *m*, then all links in *L*′ = {*L*_*i*_, …, *L*_*i*+*n*−*m*_} are assigned to *S*_*i*_. As the operation assumes that the copy number in *S* and all segments reachable from it is of the value 1, a Hamiltonian path will follow only one of the links from each of the copies. Thus *n* − *m* links in *s* are spurious, i.e., they represent sequence overlaps, but do not connect segments originating from the same region of the target sequence. Assigning *n* − *m* + 1 links to each copy as described still allows one to use any combination of *m* out of *n* links. For example, say *n* = 3 and *m* = 2, i.e., *S* = {*S*_1_, *S*_2_}. One of the three elements of *L* = {*L*_1_, *L*_2_, *L*_3_} must be spurious, i.e., not present in the correct assembly path, but we don’t know which one. Therefore, when traversing the graph, it must still be possible to follow any of the combinations of two *L* elements (*L*_1_, *L*_2_), (*L*_2_, *L*_3_) and (*L*_1_, *L*_3_). By the first iteration, *i* = 1, we assign to *S*_*i*_ = *S*_1_ the links *L*_*i*_ = *L*_1_ and *L*_*i*+*n*−*m*_ = *L*_1+3−2_ = *L*_2_. Similarly, when *i* = 2, the links *L*_2_ and *L*_3_ are assigned to *S*_2_.

### Linear paths

For *ω* ∈ {B, E}, let *λ*(*s*, *ω*) be the number of links of the *ω*-end of the segment *s*. If *λ*(*s*, B) = *λ*(*s*, E) = 1, we call *s* an *internal segment*.

A *linear path* is a path which starts from a segment *s*_*α*_, traverses the graph from the end *ω*_*α*_ of *s*_*α*_, such that *λ*(*s*_*α*_, *ω*_*α*_) = 1, then follows only internal segments, and enters the last segment *s*_*β*_, from an end *ω*_*β*_, such that *λ*(*s*_*β*_, *ω*_*β*_) = 1. Note that it is not required that *s*_*α*_ or *s*_*β*_ are internal segments.

RGFA provides a method for enumerating all linear paths in the graph starting from a segment *s* satisfying *λ*(*s*, B) = 1 or *λ*(*s*, E) = 1 and traversing in all directions with single links. By bookkeeping visited nodes, it is taken care that all paths are enumerated only once.

The segments in a linear path can then be collapsed into a single segment which represents exactly the same sequence as the original path. This is obtained by including the sequence of the segments in the path, either in forward or reverse direction, depending on the ends at which the traversal enters a segment (for details see Myers (2005)). After merging is completed, references to the segments at the extremities of the path are updated to refer to the merged segment.

RGFATools provides additional features for path merging, such as tracking the list of segments merged (or tag), as well as their position in the merged segment (introducing a custom mp tag), thus reducing the assembly problem in practice.

### Enforcing mandatory links

The problem of finding a Hamiltonian path is NP-hard (Karp, 1972). However, it is possible to easily recognize some edges, which are not compatible with such a path.

Assume that each connected component in the graph is a different molecule (e.g., chromosome) and that the ends of the molecule are known (i.e., there are two sequences with no links on one side; or the molecule is circular). If a segment end *e* has a single link ℓ, connecting it to a segment end *e*′, ℓ must be present in any Hamiltonian path (it is mandatory). Any other link of *e*′ cannot be part of a Hamiltonian path and can be deleted from the graph (it is superfluous). In a similar way, any link of a segment to itself is superfluous, except when it is the only link of the segment.

RGFATools provides a method which detects all mandatory links in the graph and removes all superfluous links.

### Random orientation of invertible segments

Consider a segment *s* which in both its ends *e* and *e*′ has links to the same two segment ends *f*∉{*e*, *e*′}, *f*′∉{*e*, *e*′}, *f* ≠ *f*′. We call this segment an *invertible segment*. For an invertible segment it is not possible to determine the orientation of *s* with respect to *e* and *e*′. In some cases it may still be preferable to obtain a single sequence by orienting the invertible segment randomly.

RGFATools provides a method to randomly orient invertible segments keeping track of the coordinates of the possible inversion in a custom integer array field rn. An invertible segment *s* can be written as *uvu*′, where *u* is the longest prefix of *s* such that *u*′ is the reverse complement of *u*. The coordinate of the possible inversion when randomly orienting *s* are also those of *v*. Therefore, if *s* = *wxw*′ is a merged segment, composed of the original segments *w*, *x*, *w*′, such that *w*′ is the reverse complement of *w*, then using the tracking information stored during the merging, the coordinates of the inversion stored are also those of *x*.

## Results

### Easy parsing and editing of GFA graphs

We have developed an implementation of the proposed specification of the “Graphical Fragment Assembly” (GFA) format in the Ruby programming language.

The interface was designed with the aim of clarity and user-friendliness. For example, adding a new custom optional field to a record only requires to set its value; if the field does not exist yet in the record, the corresponding setter and getter methods are automatically created by exploiting the metaprogramming features of Ruby.

Code based on RGFA is simple and often readable even for non-rubyists. The following code snippet, for example, loads a GFA graph from a file, and outputs a table of segment names and their lengths.

gfa = RGFA.from_file("graph.gfa") 
gfa.segments.each{|s| puts(s.name + "\t" + s.length)}

Besides the basic operations, such as adding, renaming or deleting elements, also more complex operations are implemented in RGFA, such as the duplication of segments and the merging of linear paths. The expressiveness of Ruby, combined with the interface design principle mentioned above, allow the user to conveniently code further complex operations. The following single statement, for example, removes all segments with a coverage less than 10 ×, as well as all isolated segments with a sequence shorter than 200 bp:

gfa.rm(gfa.segments.select {|s| (s.coverage < 10) or 
                                (s.connectivity == [0,0] and 
                                 s.length < 200)})

The installation of RGFA and RGFATools is based on the standard Ruby packages management system *rubygems* and is thus automatized. There are no dependencies, and the code can run on all machines on which Ruby (version ≥2.0) is installed. The code of RGFA is designed to be easily maintainable and is accompanied by a thorough test suite. This feature is particularly important, as the GFA specification is still under development (e.g., a new GFA version, 2.0, has been recently proposed) and future changes in the specification will likely require modification of RGFA.

### Comparison of GFA graphs and generation of edit scripts

The assembly graph often requires further intervention, in order to provide a complete sequence. Manual editing can for example be done using Bandage (Wick et al., 2015), which allows one to modify a graph by interacting with its graphical user interface. However, Bandage does not record the operations applied to the graph. Also if automated graph manipulation tools are applied, it can be interesting to examine how they exactly changed the graph.

Although GFA is a line-based text format, parsing is required, in order to recognize whether a difference in the text is significative or not. For example, if two segments just differ by the order of the optional fields, they are equivalent to each other. However, general purpose text-comparison tools such as diff would not be able to recognize this equivalence.

For this reason, we implemented a tool, GFAdiff, based on RGFA. The tool parses and compares to each other two GFA files. Differences in all record types are detected. Thereby, the comparison is granular: e.g., the comparison of segments with the same name is done at the level of fields. The set of differences is output in a format similar to that of the diff tool. Alternatively, using the option -script, GFAdiff is able to generate an RGFA-based Ruby script, which, when applied to the first GFA graph generates the second one.

### Case study: assembly of a repetitive fosmid insert

Functional screening of fosmid-based metagenomic libraries is a powerful method to analyze environmental DNA and discover new genes coding for an enzyme of interest (Martínez & Osburne, 2013).

In a collaborative project (manuscript under preparation), the goal was to sequence the insert of selected fosmids from a metagenomic library. Sequences were obtained from single clones using multiplexed paired-end sequencing on an Illumina MiSeq platform, with a read length of 2 × 300 bp, and an insert size of ≈ 400 bp. We applied standard preprocessing methods to the sequencing data.

**Figure 1:**
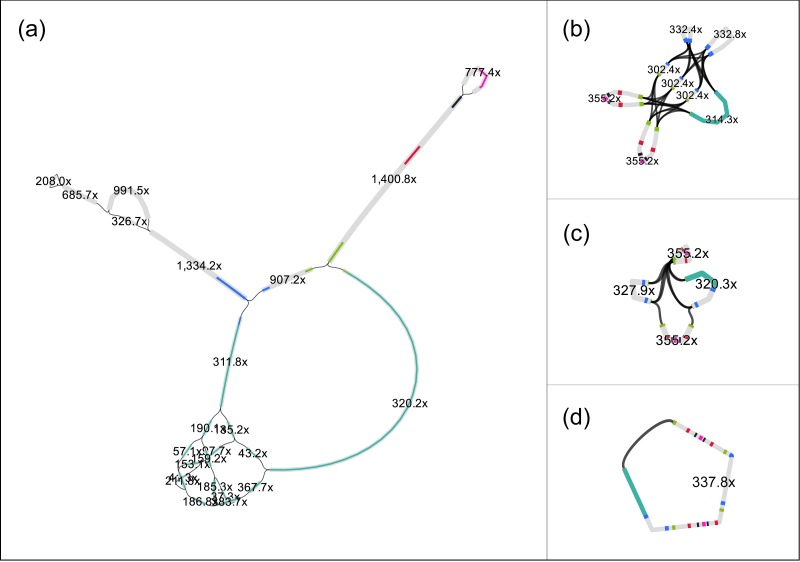
The original assembly graph of the fosmid data as delivered by SPAdes (A) and the simplifications obtained by RGFATools (B, C, D). The figure was prepared using Bandage (Wick et al., 2015).

Despite the short target sequence lengths (vector 8 kbp and insert up to 40 kbp), assembling the fosmids proved to be challenging. For example, after preprocessing, the read set of fosmid F1 (estimated length ≈ 47 kbp) contained 40⋅10^3^ sequences, with an average length of 400 bp (coverage ≈340 ×). We applied different assemblers, but, despite the high coverage and the conservative preprocessing, none was able to completely assemble the fosmid sequence. For example, SPAdes (Bankevich et al., 2012) delivered 18 contigs with a total length of 21.7 kbp. The assembly graph of SPAdes (Fig. 1A) shows that the contigs have a read coverage between 37 × and 1, 400 ×, which indicate that the insert has likely a repetitive structure. Aligning the contigs to the vector sequence (pCC1FOS, GenBank acc. EU140751) by BlastN, shows (Fig. 1A, cyan) that most of the vector sequence is contained in a contig, with length 6.9 kbp and coverage 320.2 ×.

We applied RGFATools to solve the repetitive structure of the insert. First, segments were multiplied according to their copy numbers, which were computed from the expected coverage (340 ×). Then a minimal coverage filter of 60 × was applied and p-bubbles remaining in the vector sequence were eliminated, by discarding the path with lower coverage. After merging the remaining linear paths in the graph, we obtained a graph with eight segments and 32 links (Fig. 1B).

Finally, we enforced mandatory links and randomly oriented invertible segments in a loop, merging linear paths after each operation. After the first two iterations, the graph contained only four segments and six links (Fig. 1C). The third iteration yielded a single segment (Fig. 1D) of 46.3 kbp.

### Case study: identifying CRISPRs in de Bruijn graphs

Clustered Regularly Interspaced Short Palindromic Repeats (CRISPRs) are a common feature of prokaryotic genomes. Each locus contains short conserved directed repeats (24–47 bp) separated by unique spacers (26–72 bp) (Sorek, Kunin & Hugenholtz, 2008).

Tools such as PILER-CR (Edgar, 2007) are able to detect CRISPRs in an assembled sequence. However, obtaining an assembled sequence is not always possible, in particular for applications such as metagenomics. Here the method of Ben-Bassat & Chor (2015) is more appropriate as it can identify CRISPRs in partial overlap graphs obtained from readsets.

**Figure 2:**
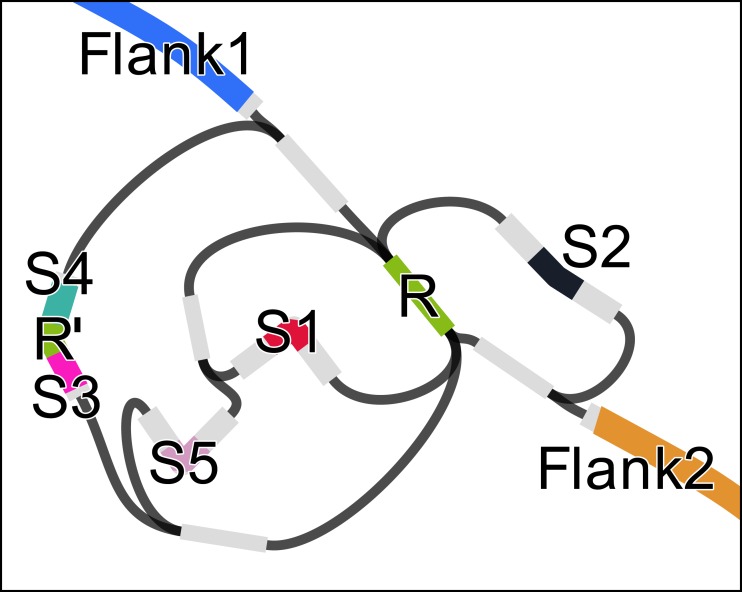
Assembly graph of a 2.3 kbp region of the genome of *Acinetobacter* sp. ADP1 containing a CRISP with 6 instances of a 28 bp repeat (R), one of which containing a mismatch (R’) and 32 bp long spacers (S1–S5). The figure was prepared using Bandage (Wick et al., 2015).

We used RGFA to implement (in less than a day) an algorithm to find potential CRISPR signatures in a de Bruijn graph with *k* < |*R*| + 1 where |*R*| is the minimum length of a CRISPR repeat (|*R*| = 28 in this case study). If a segment represents a CRISPR repeat, then it will have a higher copy number compared to its adjacent segments, and graph structure in the surroundings will typically present several branches (as in Fig. 2). Let |*s*| be the sequence length and *cn*(*s*) be the copy number of a segment *s* (which can be computed from its *k*-mer count). Let *cmin* be the minimum repeat count (default: 3), *lrmin* and *lrmax* the minimum and maximum repeat length, and *lsmin* and *lsmax* the minimum and maximum spacer length. To identify CRISPRs, we start a depth-first traversal from all segments *r* such that *cn*(*r*) > *cmin* and *lrmin* ≤ |*r*| ≤ *lrmax*. Each segment *s* is traversed at most *cn*(*s*) times. Let *u* be the sequence of the path, non including *r*. The traversal is interrupted if |*u*| > |*r*| + *lsmax*⋅2 (terminus) or the path arrives at *r* again (circle). If *cn*(*s*) − 1 circles and 2 termini are found, the CRISPR candidate is output.

Let *r*_1_, …, *r*_*n*_ be the ordered set of repeat instances in a CRISPR with *n* repeats. Let *s*_*i*_ be the spacer after *r*_*i*_. Some inexact repeats may be present. If *r*_*i*_ is inexact and *i* = 1 or *i* = *n*, the instance will not be found. If 1 < *i* < *n*, then the length of the path between *r*_*i*_ and *r*_*i*+2_ will be |*s*_*i*_| + |*r*_*i*+1_| + |*s*_*i*+1_|. For this reason, we allow the traversal to continue up to a path length |*r*| + *lsmax*⋅2. In case the distance is larger than |*r*|, we indicate in our output, that an inexact repeat instance is probably present.

In a preliminary test of the algorithm, we constructed a de Bruijn graph from regions of the *Acinetobacter* sp. ADP1 genome (Refseq acc. NC_005966) 1 kbp around the three CRISPR arrays predicted by PILER-CR with default parameters. We simplified the de Bruijn graph by merging linear paths using RGFA and analyzed the resulting graph using the algorithm sketched above. The RGFA-based program was able to correctly identify the CRISPR array in all three regions.

The algorithm was tested using exact copy numbers and assuming no erroneous *k*-mers and paths are present in the graph. Applying it to a graph constructed from real sequencing reads will be investigated in the future.

## Discussion

The current assembly algorithms use two different flavors of assembly graphs, de Bruijn and string graphs. Besides a different construction, both graph flavors represent the same information: the set of possible assemblies of a set of sequencing reads. A de Bruijn graph is equivalent to an overlap graph, where nodes are the *k* − 1-mers and edges are the overlaps of length *k* − 2. Thus, after the construction and simplification, both graphs can be output using a common format.

Most assemblers provide some kind of output of the final assembly graph. This can be useful for further sequence analysis, as it contains information on how the contigs relate to each other. However, the graph must be output in a standard format, in order to allow using the graph by other programs, besides the sequence assembler, which constructed it.

A first effort to design such a format was FASTG (Jaffe et al., 2012). This is an extension of the FASTA format, a choice which is motivated by the fact that most assemblies are output as MultiFASTA collections of contigs. However, FASTG presents several issues and, although proposed already in 2011, its adoption was very slow (Melsted & Crusoe, 2014). For example, in FASTG sequences are represented on edges of the graph; This complicates operations such as changing a sequence to its reverse complement (Li, 2014).

GFA, the “Graphical Fragment Assembly” format was designed in order to cope with the shortcomings of the FASTG format (Li, 2014). It is already mature and is used by some early adopters (GFA Format Specification Working Group, 2016), despite the fact that the specification is not yet finalized. Similarly to other popular formats in sequence analysis, such as SAM (Li et al., 2009), GFA is a text-based tab-separated values format with single-line records (headers, sequences, overlaps, etc…). Thus, GFA files can be processed by line oriented text-based utilities, such as the POSIX tools sort or grep. However, more complex operations require to parse the records and construct the graph. Therefore, specialized tools are needed.

ABySS (Simpson et al., 2009), BFGraph (http://github.com/pmelsted/bfgraph), miniasm (http://github.com/lh3/miniasm) and McCortex (Iqbal et al., 2012) allow the output of their assembly graphs in GFA format. LA2gfa (http://github.com/jts/daligner) constructs a GFA graph from the results of Daligner (Myers, 2014), a local aligner for PacBio reads. The tool vg (http://github.com/vgteam/vg), implementing sequence variation graphs also allows output in GFA format. The format conversion tool gfatools (http://github.com/lh3/gfatools) allows to convert the graphs of Velvet (Zerbino & Birney, 2008), SPAdes, Soapdenovo (Luo et al., 2012), and SGA (Simpson & Durbin, 2012) to GFA. Manual GUI-based editing of a GFA graph is possible using Bandage (Wick et al., 2015). However, neither Bandage, nor any other of the mentioned tools offer scripting capability or an application programming interface to their GFA implementation.

RGFA is an implementation of GFA in the scripting language Ruby providing an API, which allows to read, validate, write and manipulate GFA files and graphs. It is available under a free software license (http://www.isc.org/downloads/software-support-policy/isc-license/).

Another implementation of the GFA specification in the Ruby programming language has been developed in parallel with our software: the gfa library (https://github.com/lmrodriguezr/gfa). Its implementation appears to be rather incomplete; e.g., the current version (0.1.2), does not provide an interface for simple graph operations, such as efficiently enumerating the links which connect a given segment. Complex operations, such as linear path detection and merging are not available. Furthermore, the most recent changes of the GFA specification, such as the support of JSON types, have not yet been reflected in the gfa library.

RGFA graphs can be converted into an RGL (Ruby Graph Library) graph object (https://github.com/monora/rgl). This is a generic framework for graph data structures and algorithms, offering e.g., generic traversal operations, and graphical output using GraphViz (http://www.graphviz.org/). RGL is not specific for assembly graphs and some peculiarities of the GFA graphs, such as the double strandedness of the segments, and the different kind of edges do not have a direct representation in the framework (e.g., two vertices, with both orientations, must be added for each segment). RGL graphs respecting some conventions can be converted to RGFA graphs. This allows for interoperation of the different libraries, despite some limitations: a graph file can be parsed using the gfa library, converted to an RGL graph (losing part of the information, such as optional fields, segment sequences, containments and paths), then to an RGFA graph.

RGFA is based on the Ruby programming language. Conceived as an interpreted scripting language, its performance is often not sufficient for very large datasets. We tested the performance on a medium sized dataset, consisting of a de Bruijn graph from a bacterial genome, including 3.5 million segments and about the same amount of links. The memory footprint of interpreted object-oriented languages is often a problem. However, for parsing the GFA file and creating its memory representation, the memory peak of our library was only about 2.3 × larger than the memory peak of Bandage (4.3 Gb), which is implemented in C++. Also, our library was able to simplify the graph in acceptable time. Nevertheless, for similar applications, speedup could be achieved by implementing some of the time-critical algorithms as C extensions for Ruby. These would also allow to support multithreaded parallel processing, as extensions are not limited by the Global Interpreter Lock.

In contrast to a graphical editing program, such as Bandage, RGFA allows to create manipulation pipelines, which can then be applied to several graphs or their connected components in a unified way without manual interference. Furthermore, the simple RGFA syntax is designed to be understood even by readers not proficient in the use of Ruby. Thus RGFA can be used for creating scripts exactly documenting custom editing of assembly graphs, which are otherwise often only vaguely described in the method section of papers.

## Additional Information and Declarations

### Data Availability

The following information was supplied regarding data availability: Github: https://github.com/ggonnella/rgfa.
